# Disentangling the Contributions to the Proton Magnetic Shielding in Carbon Nanohoops and Nanobelts: Evidence for a Paratropic Belt-Current

**DOI:** 10.1021/acs.jpclett.0c02261

**Published:** 2020-08-17

**Authors:** Francesco F. Summa, Guglielmo Monaco, Lawrence T. Scott, Riccardo Zanasi

**Affiliations:** † Department of Chemistry and Biology “A. Zambelli”, Università degli Studi di Salerno, via Giovanni Paolo II 132, Fisciano 84084, SA, Italy; ‡ Department of Chemistry, University of Nevada, Reno, Nevada 89557-0216, United States

## Abstract

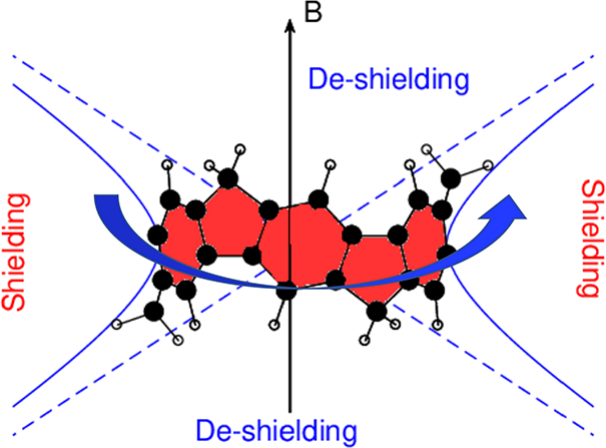

The proton NMR magnetic shieldings of the recently synthesized *D*_3d_ isomers of methylene-bridged [6]cycloparaphenylene (MB[6]CPP) and [12]cyclophenacene hide in themselves the effect of a global paratropic current around the nanobelts, which is induced by a magnetic field parallel to the main symmetry axis of the molecules. The effect is particularly pronounced for the methylene protons of MB[6]CPP, especially for those facing inside the nanobelt. The small experimental chemical shift difference of only 0.2 ppm is incompatible with the separation of the signals caused by the belt curvature, which, by itself, is calculated to be larger than 1 ppm, with both signals shifted upfield with respect to the position detected for the nanobelt. A careful dissection of the proton magnetic shielding in terms of molecular orbital contributions, has permitted a quantitative assessment of the genuine effect on each different proton caused by a substantial paratropic belt-current, which brings all the signals in nice agreement with the experimental spectra.

The [*n*]cycloparaphenylenes ([*n*]CPPs) are the shortest cross-section of an [*n*, *n*] armchair carbon nanotube ([*n*, *n*]CNT), and since their first synthesis in 2008 by Jasti et al.,^1^ they have attracted a lot of attention for their fascinating structure and properties,^2^ as well as for their potential use as seeds from which to grow carbon nanotubes of uniform diameter and single chirality.^3,4^ The radially oriented p orbitals and distorted geometries constitute their major features. The canted benzene rings around the macrocycle display torsional angles θ that are related to the CPP size in such a way that the smaller the *n* the smaller the θ.^2,5,6^ The consequent increased conjugation offers one possible explanation for the observed narrowing of the HOMO–LUMO gap as *n* decreases, which lends unique optoelectronic properties to CPPs.^7^

One way to reduce θ to a nearly vanishing value is to rigidly close the bays between neighboring aryl rings, for example, by means of an ethylene or a methylene bridge, which transforms [*n*]CPPs to isomers of [2*n*]cyclophenacenes or methylene-bridged (MB) [*n*]CPPs. Weighable amounts of such beautiful nanobelts have been recently obtained by the Itami group, who synthesized the *D*_3d_ isomers of [12]cyclophenacene (**1**)^8,9^ and MB[6]CPP (**2**).^10^

Rather interestingly, both nanobelts **1** and **2** are predicted to sustain a global paratropic current around the belt in response to a magnetic perturbation parallel to the main symmetry *z*-axis. This is a consequence of the HOMO and LUMO symmetries (see the Supporting Information for details) whose direct product matches exactly the symmetry of the rotation *R*_*z*_.^11,12^ This behavior is typical of antiaromatic species, and a significant paratropic contribution to the current density induced by a parallel magnetic field is expected to occur, as previously reported for [10]cyclophenacene^13^ and ultrashort single-end-capped [5,5] carbon nanotubes.^14^

Thanks to Itami’s newly reported and diversified experimental data, the possibility to validate such a prediction can now finally be accomplished. In particular, we will show the major imprint of the paratropic current on the ^1^H NMR chemical shifts of these nanobelts.

A powerful tool to detect and quantify delocalized currents, either diamagnetic (aromatic) or paramagnetic (antiaromatic), is provided by the so-called current strength, or current susceptibility,^15,16^ which provides the net current strength crossing a plane perpendicular to a selected bond in a molecule. By definition, only delocalized currents can give a contribution^17^ (see the Supporting Information for details). Current strengths calculated at the B97-2/6-311+G(2d,p)//B97-2/6-31G(d) level,^18−24^ induced in **1** and **2** by a magnetic field parallel to the main symmetry axis, are shown in Figure 1.

**Figure 1:**
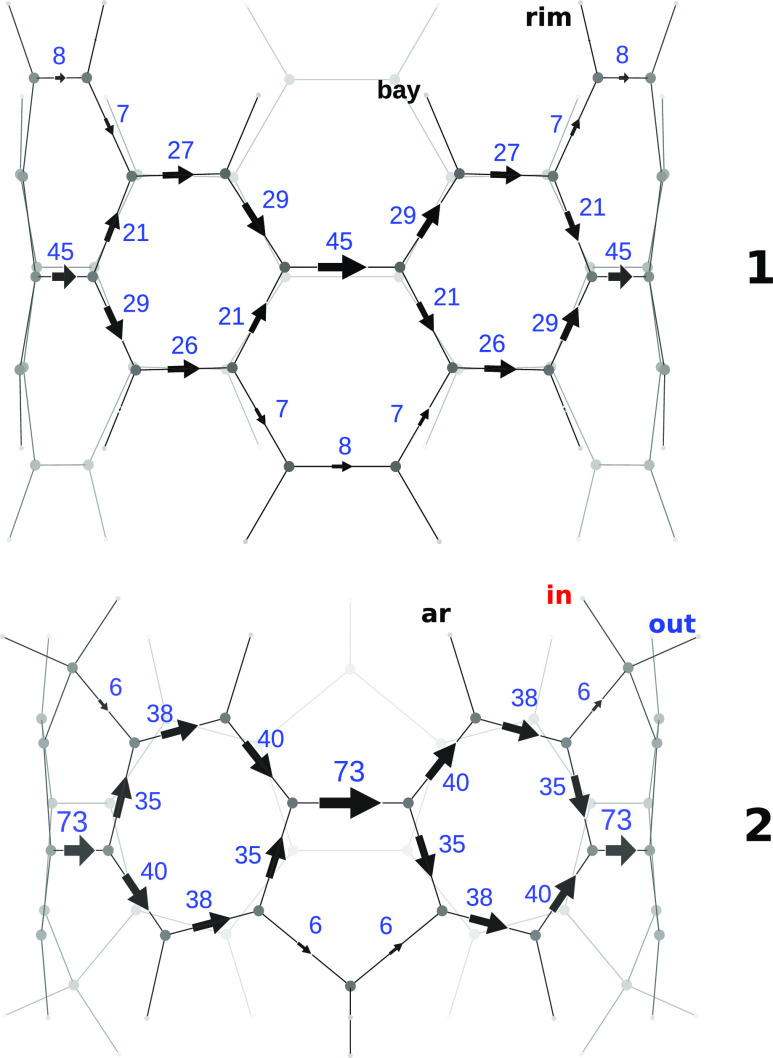
Net C–C bond current strengths for a magnetic field parallel to the main symmetry axis and pointing from bottom to top. Values aside each arrow represent the percentage relationship with respect to the benzene current strength. Circulation from left to right are globally paratropic/antiaromatic.

Origin independence of the current density has been ensured by using the continuous set of gauge transformations method with atomic size adjustments determined by the bond critical points of the electron density distribution (CSGT-BCP).^25^ As can be observed, in both molecules the current flow is paratropic and bifurcates and gathers around the six-membered rings of the imbedded cycloparaphenylene nanohoop. It can be noticed that both macrocycles have a quinoidal resonance structure along the cycloparaphenylene nanohoop that provides two pathways, an upper and a lower one, both corresponding to a [4*n*]annulene. From this perspective, the result is consistent with Hückel’s rule for antiaromaticity. It is conceivable that the larger the weight of the quinoidal resonance structure, the larger would be the [4*n*]annulene behavior.^14^ In **1** the current strength reaches 45% of the magnitude of the benzene current strength (BCS), whereas in **2** the current is much stronger, reaching 73% of BCS, which is in agreement with the larger current expected for those belts hosting a fully-Clar structure,^14^ for which the weight of the quinoidal resonance structure is higher. Further maps calculated adopting the B3LYP^26^ and M06-2X^27^ density functionals providing essentially the same picture can be found in the Supporting Information.

As recently shown,^25^ volumetric integration of the shielding density function, i.e., the CSGT-BCP current density times an appropriate geometrical factor, provides accurate magnetic shielding constants (σ_*i*_) especially when *i* indicates a nucleus independent position. For a nucleus, σ_*i*_ can be accurately converted to the corresponding NMR chemical shifts δ_*i*_ relative to TMS by using a suitably chosen reference molecule;^28^ see the Supporting Information for details on transforming σ’s to δ’s. Predicted ^1^H NMR chemical shifts are reported in Table 1. These are found to be in fairly good agreement with the available experimental data. Positions and relative shifts are nicely reproduced. Only a few small discrepancies can be observed, i.e., for the M06-2X H_in_ of **2**, which is predicted a little upfield and the B3LYP and B97-2 H_ar_ of **2**, which are predicted a little downfield.

**Table 1:** CSGT-BCP ^1^H NMR δ’s in ppm at the DFT/6-311+G(2d,p)//DFT/6-31G(d) Level

DFT	**1**-H_rim_	**1**-H_bay_	**2**-H_ar_	**2**-H_in_	**2**-H_out_
B3LYP	7.55	8.39	8.08	4.01	4.21
M06-2X	7.45	8.35	7.82	3.76	4.21
B97-2	7.56	8.40	8.11	4.08	4.22
expt^9,10^	7.51	8.26	7.86	4.09	4.29

Putting aside for now the aromatic protons, which will be discussed later, we have found particularly interesting the positions of the signals for the methylene protons in **2**. According to the *anisotropy effect*(29) due to the paratropic belt current, the methylene protons overlooking the current should experience a deshielding effect higher than that felt by the protons facing the outside of the belt; see Figure 2 for a very rough representation. Contrary to expectations based on this simple picture, however, both the experimental data and the calculated chemical shifts show H_in_*more shielded* than H_out_ by 0.20 ppm.^10^

**Figure 2:**
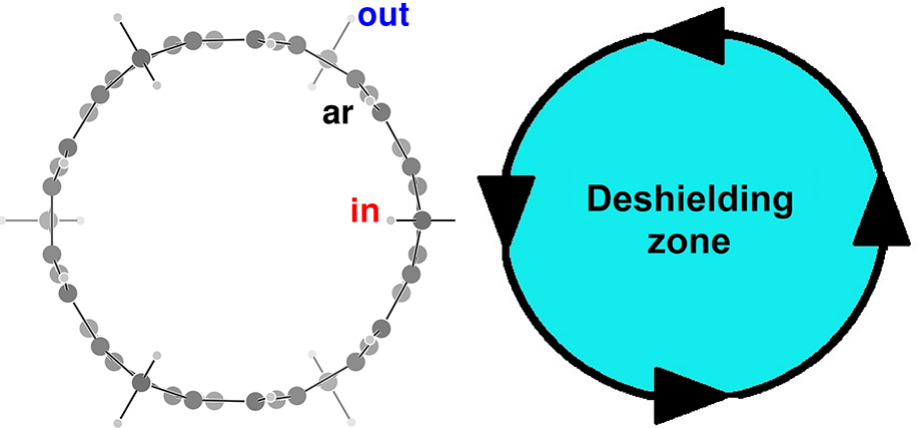
Left: top view of **2**. Right: schematic representation of the paratropic current flowing in a tiny wire having the nanobelt radius.

Of course, other factors superimposed on the paratropic belt current must be taken into account, as, for example, the diatropic currents induced on the distorted atomic scaffold by a perpendicular magnetic field. These currents are predicted to be mainly local to the benzene rings, with sizable portions flowing on the methylene groups as a consequence of the hyperconjugation between the aliphatic C–H bonds and the aromatic π-system; see the Supporting Information for more details.

To deconvolute this rather complex situation, we have explored the consequences of cutting the belt to switch off the paratropic current. In addition, to study the influence of the curvature, we have considered planar fluorene (**3**), folded fluorene (**4**), and a half of the nanobelt (**5**), as shown in Scheme 1. The geometries of **4** and **5** are taken by cutting out the fragments from **2** without further geometry optimization, apart from adding hydrogen atoms to saturate broken bonds.

**Scheme 1:**
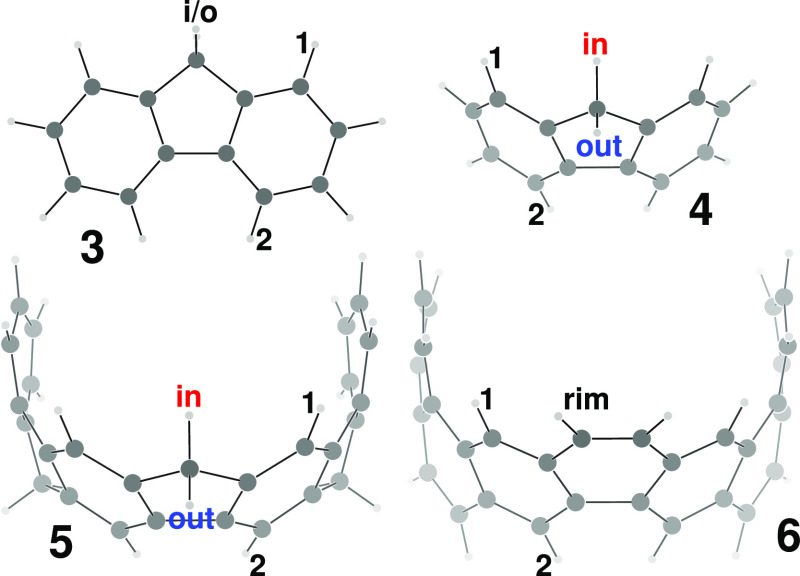
Fluorene **3**, Folded Fluorene **4**, Half Nanobelts **5** and **6**

Calculations of proton chemical shifts of **3**–**5** have been carried out at the same levels of theory as above. Here and in the following results are shown for the B97-2 functional; results obtained adopting the B3LYP and M06-2X can be found in the Supporting Information.

As can be observed in Table 2 passing from **3** to **4**, the bending induces effects on all the protons. The most impressive is the splitting of the methylene proton signals in opposite directions: the inner proton moves upfield by nearly 0.6 ppm, while the outer proton moves downfield by about 0.5 ppm. Ignoring for now the magnitude of this very large separation of 1.1 ppm, this corresponds to the relative position of the signals in **2**, where the inner proton is more shielded than the outer one. A second effect that can be observed is on the aromatic protons retained in **2**, both of which undergo an upfield shift ranging within 0.2–0.4 ppm. These effects can be readily explained as follows. First, looking at the folded structures, it is easy to recognize the different exposures of the methylene protons to the diamagnetic ring current of the two proximal benzene rings: H_in_ going inside the shielding zones and H_out_ going outside. Second, the decreased conjugation reduces the strength of the benzene ring current with a consequent upfield shift of the aromatic proton signals.

**Table 2:** CSGT-BCP ^1^H NMR δ’s in ppm at the B97-2/6-311+G(2d,p) Level

mol	H_ar1_	H_ar2_	H_in_	H_out_
**3**	7.56	7.87	3.78	3.78
**4**	7.39	7.50	3.18	4.28
**5**	7.27	7.29	3.01	4.11

This picture is nicely consolidated in **5**, where the aromatic proton signals get closer to each other and move a little further upfield and the methylene proton chemical shifts reach presumably their final values in the absence of the paramagnetic belt current, 1.1 ppm apart.

Next, to see if any computationally less intensive method could be found to estimate as close as possible the effect of the paramagnetic belt current, we have considered the few electron model by Steiner and Fowler,^11,12^ calculating the orbital contributions to the current strength for the bond connecting the benzene rings along the cycloparaphenylene nanohoop of **2**, induced by a magnetic field parallel to the main symmetry axis, due to the HOMO, HOMO–1, ..., and so on. Of course, the full orbital sum gives the value reported in Figure 1, corresponding to 73% of BCS. The hope is to find some stable value much before using only a few frontier orbitals, whose contribution to the current density would be a genuine feature of the belt. As expected, the A_2g_ HOMO alone gives a very large paratropic current strength equal to 138% of BCS, which is mainly due to virtual transitions to the LUMO and LUMO+1, both of A_1g_ symmetry. Adding the doubly degenerate E_u_ HOMO–1, the current strength remains paratropic but reduces to 65% of BCS, as might be expected since a diatropic contribution is determined by the (*x*, *y*) translational symmetry of the virtual transitions to the LUMO and LUMO+1. Adding one more occupied orbital, i.e., the A_2u_ HOMO–2, the current strength rises to 67% of BCP, from virtual transitions to higher virtual orbitals, and then it does not show any further change adding up to 12 more occupied MO’s. This nice result now allows us to confidentially estimate the paratropic belt current effects as those arising from the four HOMO, HOMO–1 (doubly degenerate), and HOMO–2 only, which provide a stable current strength that closely matches the total one. Moreover, maps of the induced current density clearly show that the flow generated by these few orbitals is fully delocalized all over the belt; i.e., it is a genuine feature of the macrocycle (see the Supporting Information for details).

The contribution to σ_*zz*_ of **2** given by HOMO, HOMO–1 (partner *x*), HOMO–2 (partner *y*), and HOMO–2 has been calculated over a grid of points forming a plane containing one methylene group (see Figure 3) and over a plane containing two opposite C–H bonds of a benzene ring (see Figure 4), as previously reported in much simpler cases.^30^

**Figure 3:**
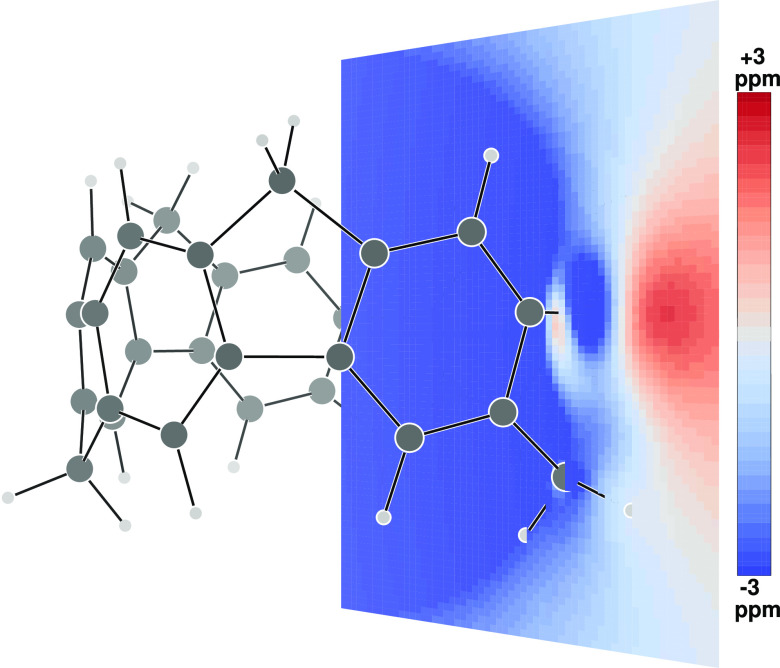
Divergent color map of the contribution to σ_*zz*_ of **2** given by HOMO, HOMO–1(*x*), HOMO–1(*y*), and HOMO–2 over a plane containing one methylene group. Only half of the plane from the molecular axis is shown.

**Figure 4:**
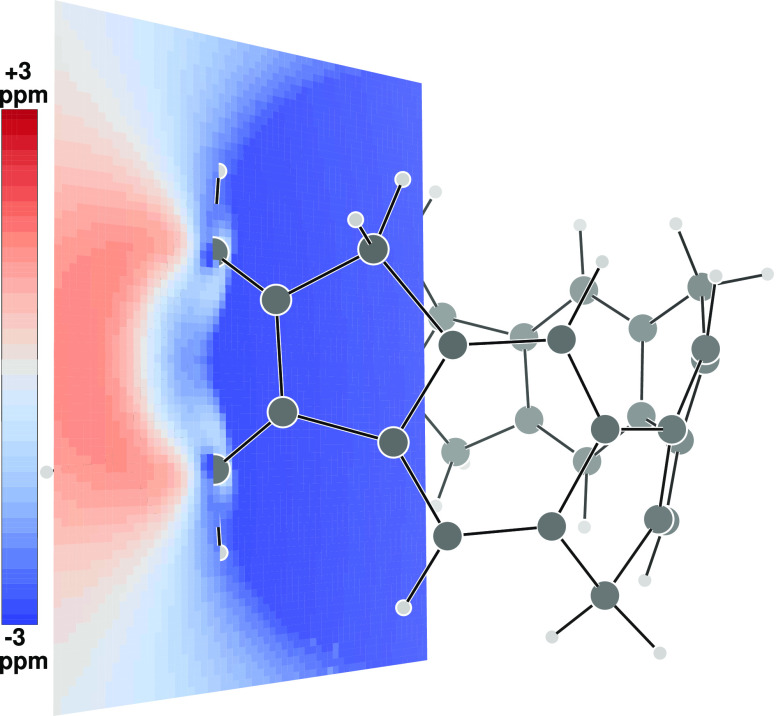
Same as in Figure 3 for a plane containing two opposite C–H bonds of a benzene ring.

As can be observed, a deshielding zone (blue) fills the interior of the nanobelt and extends a little also on the outside. The shielding zone (red) forms a kind of donut along the equator and in many aspects the two maps recall some general features already observed in the ICSS of axial molecules.^31,32^ Protons are found to lie in the deshielding zone; precisely, the contribution to σ_*zz*_ at H_in_ is equal to −3.35 ppm, while it is found to be only −0.30 ppm at H_out_ and −2.71 ppm at H_ar_.

The fact that both methylene protons are deshielded is only deceptively in contrast with a basic ring current model. Figure 5 shows the line of null shielding, separating deshielding and shielding zones, for a single infinitely thin loop of current. According to the dashed line, computed for a loop of negligible radius, i.e., the so-called dipole approximation,^29^ the protons should have shielding of different signs, but according to the continuous line (computed for a radius of 3.95 Å as indicated by the minimized geometry), they should both be deshielded.^33^

**Figure 5:**
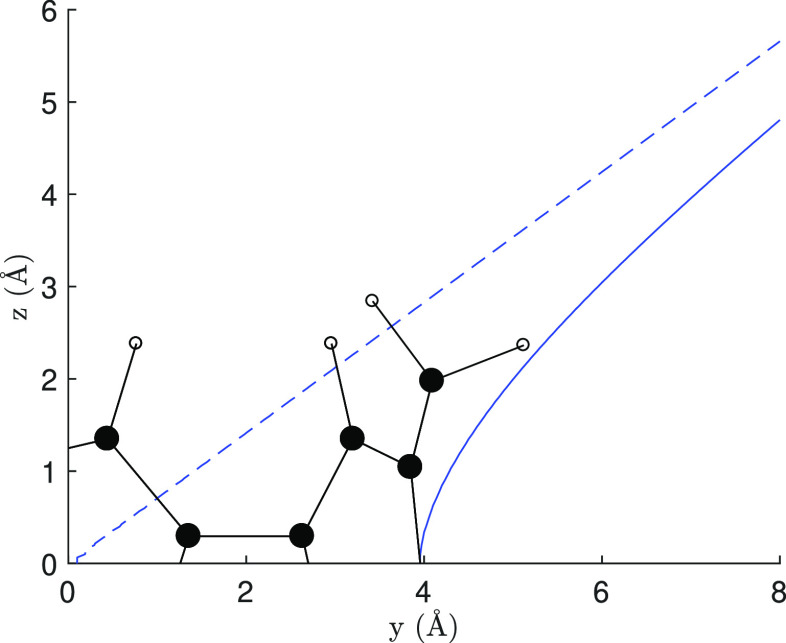
Line of null shielding computed for a loop of current (see Johnson and Bovey,^33^ eq 6) assuming a loop of negligible radius (dashed line) or for a loop of 3.95 Å (continuous line), on a plane bisecting the loop.

Since each diagonal component of the magnetic shielding tensor provides a contribution equal to one-third of its value to the average shielding constant, we can consider one-third of the absolute values of the above estimations, i.e., 0.90, 1.12, and 0.10 ppm, as the downfield contributions, due to the paramagnetic belt current, to the ^1^H_ar_, ^1^H_in_, and ^1^H_out_ magnetic shielding constants, respectively, and add them to the proton chemical shifts of **5**, listed in Table 2, which are the consolidated δ’s for **2** in the absence of the paramagnetic belt current. We obtain 8.18 ppm for H_ar_, 4.13 ppm for H_in_, and 4.21 ppm for H_out_, which compare very nicely with the B97-2 results of the intact belt reported in Table 1. In other words, the dissection worked wonderfully, providing strong evidence for the different and opposite effects due to the curvature and the paramagnetic belt current in **2**.

As far as it concerns **1**, the paramagnetic belt current effects can be disclosed using a similar strategy. In brief, we have taken one-half of the nanobelt (**6**), as shown in Scheme 1, by cutting out the fragment from **1** without further geometry optimization. Then, ^1^H δ’s have been calculated, which turn out to be 7.47 ppm for the rim protons and 8.15–8.21 ppm for the no-longer-equivalent bay protons. As before, we have assumed these values as the proton chemical shifts in the absence of the paramagnetic belt current. Actually, comparing with the B97-2 results of Table 1, a further deshielding range can be observed. Application of the few electron model^11,12^ to estimate the orbital contributions to the current strength for the bond connecting the cycloparaphenylene benzene rings of **1**, induced by a parallel magnetic field, shows that the A_2g_ HOMO alone provides a quite large paratropic current strength equal to 97% of BCS, mainly due to the virtual transition to the A_1g_ LUMO. This is more than twice the total value of 45% reported in Figure 1. The A_2u_ HOMO–1 and doubly degenerate E_g_ HOMO–2 do not provide any significant contribution to the current strength. Instead, diatropic contributions are given by the doubly degenerate E_u_ HOMO–3 and HOMO–4, which lower the current strength value to 28% of BCS. The contribution to σ_*zz*_ given by these orbitals has been calculated to be −0.41 and −0.93 ppm for the rim and bay protons, respectively. Subtracting one-third of these values from the proton chemical shifts of **6**, one obtains a final estimate of 7.61 ppm for the H_rim_ and 8.50 in average for the H_bay_, which compare nicely with the B97-2 results of the intact belt of Table 1.

The proton magnetic shielding constants of the recently synthesized *D*_3d_ isomers of [12]cyclophenacene **1**(8,9) and methylene-bridged [6]cycloparaphenylenes **2**(10) have been dissected in detail.

It is found that the effect due to the belt curvature alone would provide, in general, ^1^H δ’s shifted to high field with respect to the experimental data. Such an effect is particularly evident for the methylene protons of **2**, especially for the proton facing inside the belt, which is calculated more than 1 ppm upfield respect to the observed signal. Aromatic protons are also calculated to be shifted upfield, ranging from 0.2 ppm for the upper rim of **1** to 0.8 ppm for **2**.

Application of the few electron model^11,12^ has permitted quantitative evaluation of the effect on the proton chemical shifts of the global paratropic belt current, induced by a magnetic perturbation parallel to the main molecular symmetry axis, predicted for these kinds of macrocycles^13,14^ but never proven, until now, on the basis of experimental results. The effect of such tubular paratropic currents result in a general but different downfield shift, which brings all the calculated ^1^H δ’s into nice agreement with the experimental data. The methylene protons of the rigidified [6]cycloparaphenylene (**2**), whose NMR signals are the most affected by the two opposite effects, provide striking evidence for the presence of the paratropic belt currents.

## Supporting Information Available

Calculation details; B3LYP and M06-2X current strengths of **1** and **2**; detailed results for fluorene, folded fluorene, phenanthrene, folded phenanthrene, and half nanobelts **5** and **6** (PDF)
