# Supplementary material for: Disentangling the Contributions to the Proton Magnetic Shielding in Carbon Nanohoops and Nanobelts: Evidence for a Paratropic Belt-Current
Source: J Phys Chem Lett. 2020 Aug 17;11(18):7489–94. doi: 10.1021/acs.jpclett.0c02261 (PMC8011916; doi:10.1021/acs.jpclett.0c02261)
Supplement: Supplementary file 1 — jz0c02261_si_001.pdf [file jz0c02261_si_001.pdf]

Supporting Information

Disentangling the Contributions to the Proton Magnetic Shielding in Carbon Nanohoops and Nanobelts: Evidence for a Paratropic Belt-Current

F. F. Summa[1], *G. Monaco*[1], *L. T. Scott*[*2], *R. Zanasi*[*1]

[1] Dipartimento di Chimica e Biologia "A. Zambelli",
Università degli studi di Salerno, via Giovanni Paolo II 132,
Fisciano 84084, SA, Italy
[2] Department of Chemistry, University of Nevada,
Reno, NV 89557-0216, USA

Contents

1 Calculation details **S2**
1.1 Chemical shift S2
1.2 Current strengths S3

2 Molecules **S3**
2.1 [12]cyclophenacene D_{3d} isomer (**1**) S3
2.2 MB[6]CPP D_{3d} (**2**) S4
2.3 Fluorene (**3**) S5
2.4 Fluorene Folded (**4**) S6
2.5 FF_3 (**5**) S7
2.6 Phenanthrene (**7**) S8
2.7 Phenanthrene Folded (**8**) S9
2.8 PP_3 (**6**) S10

1 Calculation details

The calculations reported in this work are performed using three different DFT functionals: B3LYP, M062X and B972 and basis set 6-311+G(2d,p) on 6-31G(d) optimized structures at the same level of theory. The B3LYP functional [1] has been chosen as a standard, while for the B97-2 functional, we have based our choice on the results by Flaig and coworkers[2], who have shown, bench-marking hydrogen and carbon NMR chemical shifts, the good performance of the B97-2 functional for the calculation of accurate magnetic shielding constants. Since it is known that some functionals tends to overestimate the paratropic ring-current strengths of strongly antiaromatic molecules, some authors recommend the use of functionals with a larger percentage of HF exchange to avoid this kind of overestimation [3, 4]. Functionals with about 50% HF exchange yield ring currents of about the same strength as those obtained at the MP2 level. For these reasons we have performed calculations also at the M062X level of theory. Geometry optimization has been performed with the Gaussian 16 software [5]. Magnetically induced current densities and related magnetic properties have been obtained using the CSGT-BCP [6] method with the SYSMOIC package [7].

1.1 Chemical shift

Owing to experimental difficulties, absolute NMR isotropic magnetic shielding constants are not widely adopted; chemical shifts with respect to some reference compound are used instead. In particular, the chemical shift δ_{i} for a specific nucleus in a molecule relative to TMS can be obtained as [8]

$$\delta_{\mathrm{i}} = \sigma_{\mathrm{ref}} - \sigma_{\mathrm{i}} + \delta_{\mathrm{ref}} \tag{1}$$

where σ_{ref} is the computed shielding constant for the same nucleus in a reference compound, σ_{i} is the computed shielding constant for the nucleus in the molecule of interest and δ_{ref} is the experimental chemical shift for the reference compound relative to TMS. If a nucleus has similar chemical environments in the molecule of interest as well as in the reference compound and both molecule are treated at the same level of theory, then significant error cancellations occur and chemical shifts relative to TMS can be obtained to a high degree of accuracy. For aromatic protons we use C_6H_6 as the reference compound, adopting $\delta_{\mathrm{ref}} = 7.36$ ppm in $CDCl_3$ [9]. For aliphatic protons we use TMS as the reference and eq. (1) reduces to

$$\delta = \sigma_{\mathrm{TMS}} - \sigma. \tag{2}$$

Reference values of isotropic magnetic shieldings, adopted in this work, calculated with CSGT-BCP scheme [6] are given in the following table.

	**TMS**		**C_6H_6**	
DFT	^{13}C	^{1}H	^{13}C	^{1}H
B3LYP	182.269	31.250	48.464	23.965
M062X	186.897	31.398	37.990	23.496
B972	187.255	31.300	57.074	23.946

Table S1: ^{13}C and ^{1}H absolute average magnetic shielding in ppm calculated according to the CSGT-BCP scheme [6].

1.2 Current strengths

Current strengths, or current susceptibilities, turn out to be a rather convenient choice to provide a quantitative representation of the magnetically induced current flow in a molecule. [10, 11] They have been calculated by integrating the cross section of the induced current density vector (for a given orientation of the inducing magnetic field) over a plane bisecting at right angle a bond between any two atoms. When the current flowing about a bond is characterized by a substantially local vortical regime, a net current will be obtained whose strength will be negligible. Conversely, a value different from zero is a measure of current delocalization. [12]. Current strengths have been calculated by integrating the cross section of the magnetically induced current density over a squared plane of size 4×4 atomic units, perpendicular to the selected bond and crossing the bond in its middle.

2 Molecules

2.1 [12]cyclophenacene $D_{3\mathrm{d}}$ isomer (1)

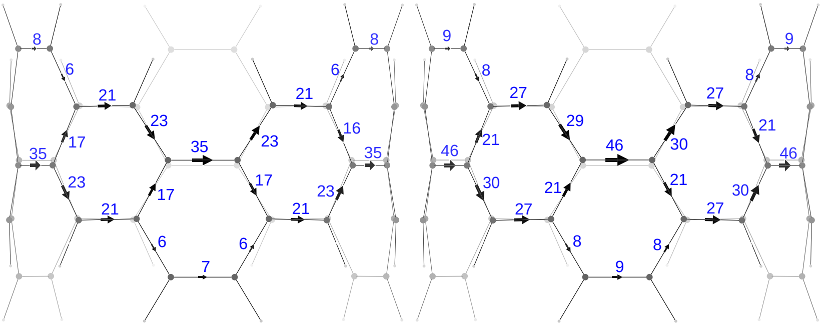

Figure S1: Current strengths of **1** calculated at M062X and B3LYP levels respectively according to the CSGT-BCP scheme [6] for a magnetic field $\mathbf{B} = \hat{\mathbf{k}}$.

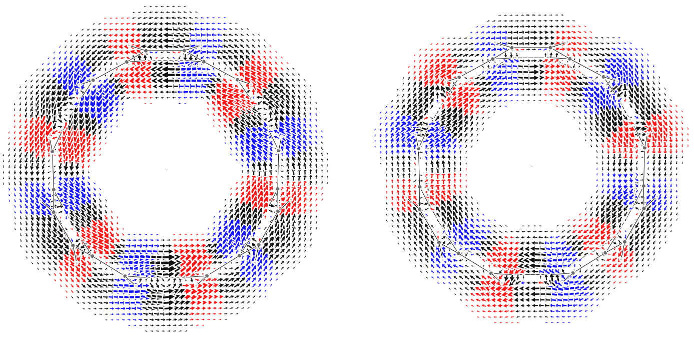

Figure S2: Paratropic and diatropic current densities of **1** generated by only taking in account the HOMO A_{2g} and HOMO-3 of symmetry E_u molecolar orbitals contributions respectively for a magnetic field $\mathbf{B} = \hat{\mathbf{k}}$.

2.2 MB[6]CPP D_{3d} (2)

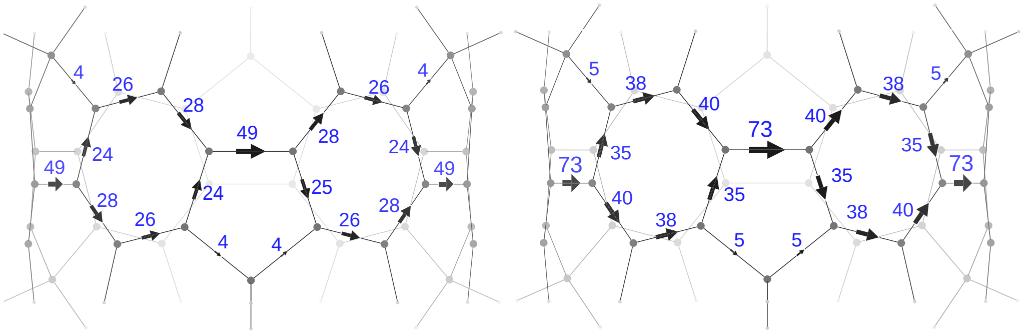

Figure S3: Current strengths of **2** calculated at M062X and B3LYP levels respectively according to the CSGT-BCP scheme [6] for a magnetic field $\mathbf{B} = \hat{\mathbf{k}}$.

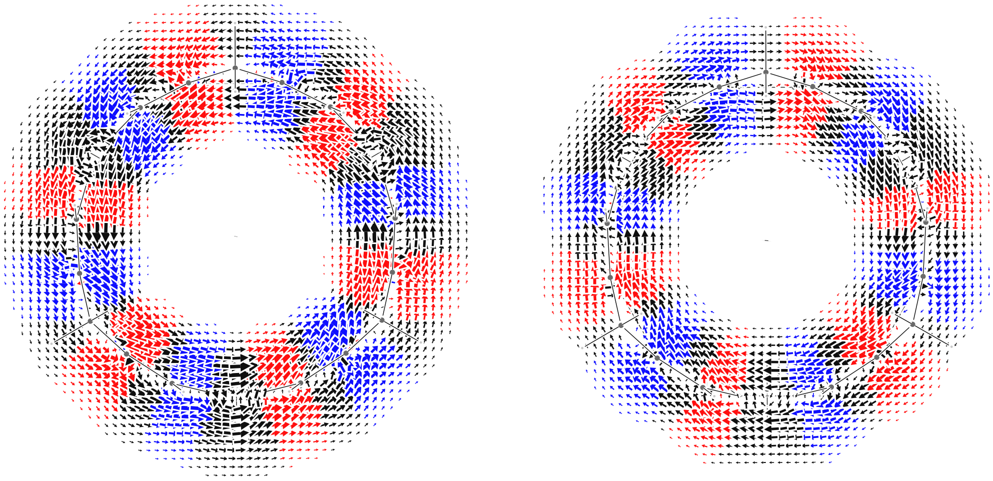

Figure S4: Paratropic and diatropic current densities of **2** generated by only taking in account the HOMO A_{2g} and HOMO-1 of E_u symmetry molecolar orbitals contributions respectively for a magnetic field $\mathbf{B} = \hat{\mathbf{k}}$.

2.3 Fluorene (3)

Molecule **3** belongs to the C_{2v} point group symmetry.

DFT	$\mathbf{H}_{ar1}$	$\mathbf{H}_{ar2}$	$\mathbf{H}_{i/o}$
B3LYP	7.52	7.81	3.69
M062X	7.55	7.89	3.85

Table S2: ^{1}H average magnetic shielding in ppm calculated according to the CSGT-BCP scheme [6] for **3** with two different functionals adopting the previous described protocol with eqs. 1 and. 2

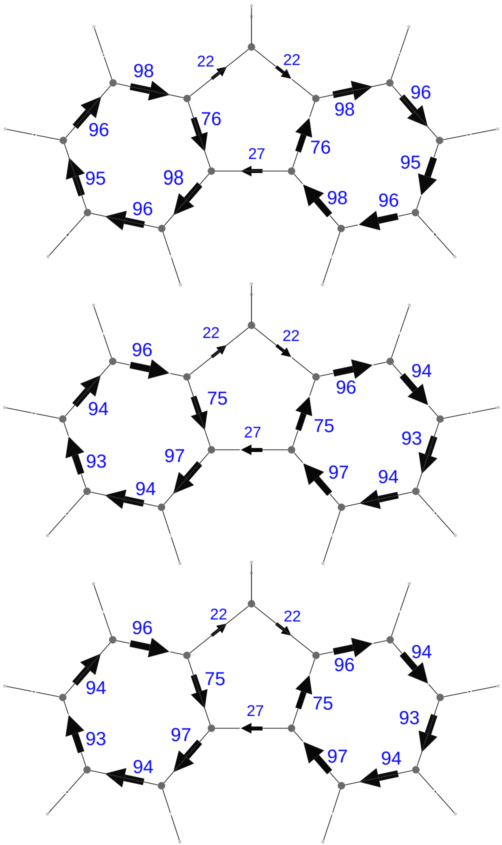

Figure S5: Current strengths of **3** calculated on the top at M062X level, on the middle at B3LYP level and on the bottom at B972 level according to the CSGT-BCP scheme [6] with a magnetic field $\mathbf{B} = \hat{\mathbf{i}}$.

As can be seen from the current strengths of **3** on the methylene group there is hyperconjugation between the aliphatic C-H bonds and the aromatic π-system.

2.4 Fluorene Folded (4)

By considering **4**, that belongs to the C_s symmetry point group, we can observe from the current strengths below that the hyperconjugation between the aliphatic C-H bonds and the aromatic π-system decrease.

DFT	$\mathbf{H}_{ar1}$	$\mathbf{H}_{ar2}$	$\mathbf{H}_{in}$	$\mathbf{H}_{out}$
B3LYP	7.37	7.50	3.13	4.26
M062X	7.44	7.59	3.36	4.35

Table S3: ^{1}H average magnetic shielding in ppm calculated according to the CSGT-BCP scheme [6] for **4** with two different functionals adopting the previous described protocol with eqs. 1 and. 2

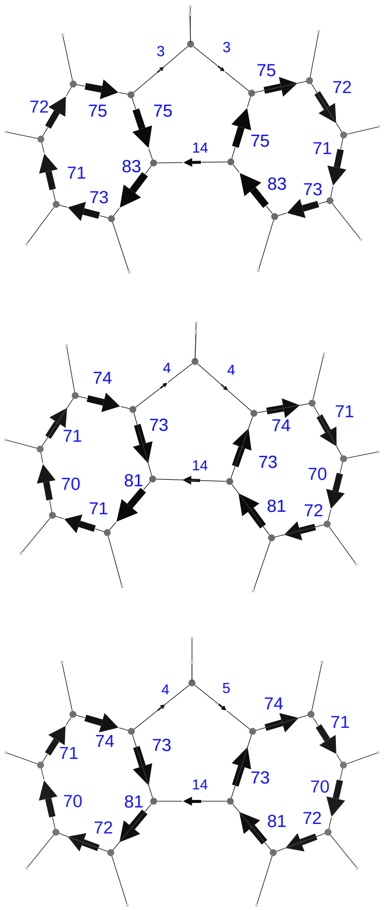

Figure S6: Current strengths of **4** calculated on the top at M062X level, on the middle at B3LYP level and on the bottom at B972 level according to the CSGT-BCP scheme [6] with a magnetic field $\mathbf{B} = \hat{\mathbf{j}}$.

2.5 FF_3 (5)

Molecule **5** belongs to the C_s point group symmetry.

DFT	$\mathbf{H}_{ar1}$	$\mathbf{H}_{ar2}$	$\mathbf{H}_{in}$	$\mathbf{H}_{out}$
B3LYP	7.26	7.28	2.96	4.10
M062X	7.36	7.39	3.16	4.19

Table S4: ^{1}H average magnetic shielding of **5** in ppm calculated according to the CSGT-BCP scheme [6] with two different functionals adopting the previous described protocol with eqs. 1 and 2.

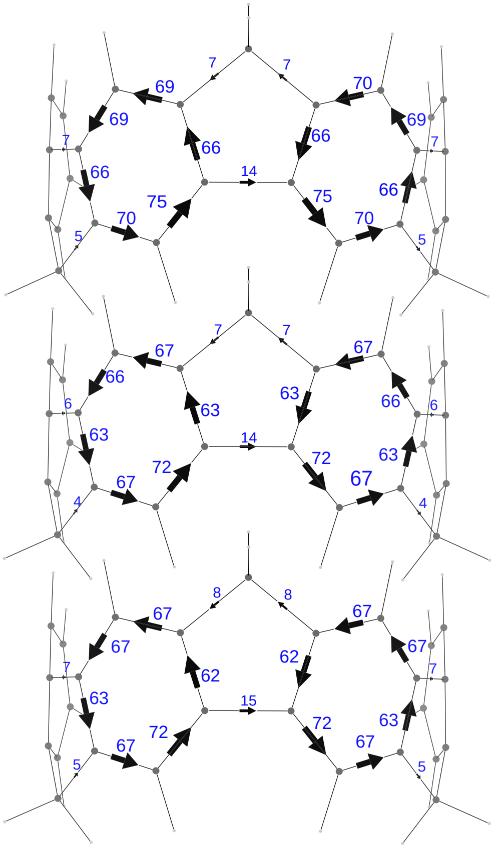

Figure S7: Current strengths of **5** calculated on the top at M062X level, on the middle at B3LYP level and on the bottom at B972 level according to the CSGT-BCP scheme [6] with a magnetic field $\mathbf{B} = \hat{\mathbf{j}}$.

2.6 Phenanthrene (7)

Molecule **7** belongs to the C_{2v} point group symmetry.

DFT	$\mathbf{H}_{bay1}$	$\mathbf{H}_{bay2}$	$\mathbf{H}_{rim}$
B3LYP	7.94	8.84	7.78
M062X	8.04	8.99	7.87
B972	7.95	8.85	7.80

Table S5: ^{1}H average magnetic shielding of **7** in ppm calculated according to the CSGT-BCP scheme [6] with three different functionals adopting the previous described protocol with eqs. 1 and. 2

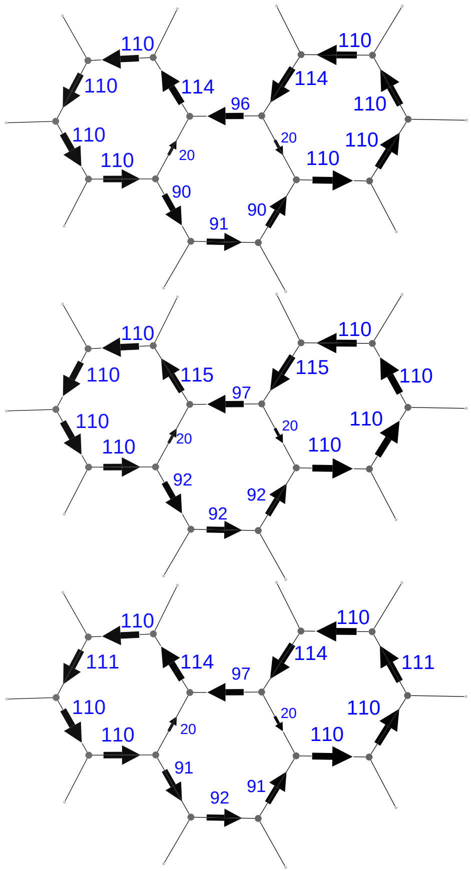

Figure S8: Current strengths of **7** calculated on the top at M062X level, on the middle at B3LYP level and on the bottom at B972 level according to the CSGT-BCP scheme [6] with a magnetic field $\mathbf{B} = \hat{\mathbf{i}}$.

2.7 Phenanthrene Folded (8)

Molecule **8** belongs to the C_s point group symmetry.

DFT	$\mathbf{H}_{bay1}$	$\mathbf{H}_{bay2}$	$\mathbf{H}_{rim}$
B3LYP	7.56	8.31	7.50
M062X	7.57	8.42	7.48
B972	7.57	8.32	7.52

Table S6: [1]H average magnetic shielding of **8** in ppm calculated according to the CSGT-BCP scheme [6] with three different functionals adopting the previous described protocol with eqs. 1 and. 2

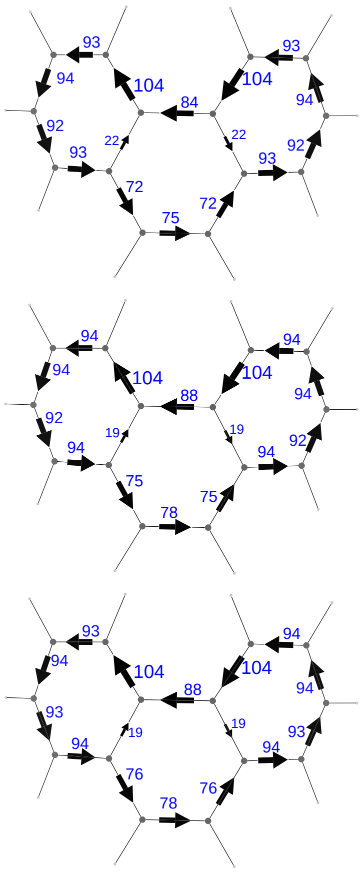

Figure S9: Current strengths of **8** calculated on the top at M062X level, on the middle at B3LYP level and on the bottom at B972 level according to the CSGT-BCP scheme [6] with a magnetic field $\mathbf{B} = \hat{\mathbf{j}}$.

2.8 PP_3 (6)

Molecule **6** belongs to the C_s point group symmetry.

DFT	$\mathbf{H}_{bay1}$	$\mathbf{H}_{bay2}$	$\mathbf{H}_{rim}$
B3LYP	8.14	8.19	7.45
M062X	8.25	8.31	7.46
B972	8.15	8.21	7.47

Table S7: ^{1}H average magnetic shielding of **6** in ppm calculated according to the CSGT-BCP scheme [6] with three different functionals adopting the previous described protocol with eqs. 1 and. 2

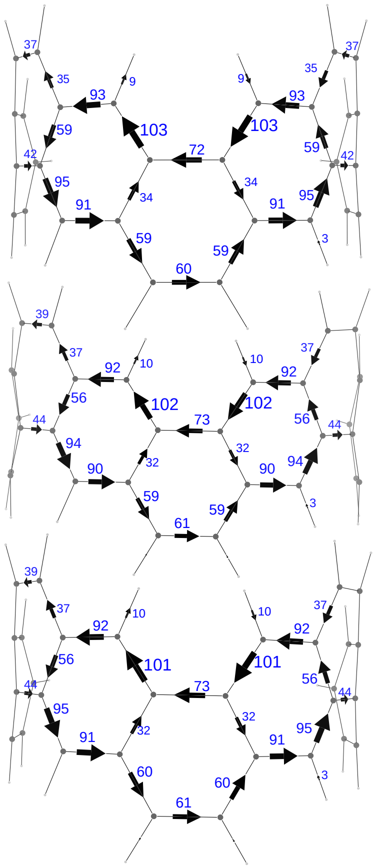

Figure S10: Current strengths of **6** calculated on the top at M062X level, on the middle at B3LYP level and on the bottom at B972 level according to the CSGT-BCP scheme [6] with a magnetic field $\mathbf{B} = \hat{\mathbf{j}}$.

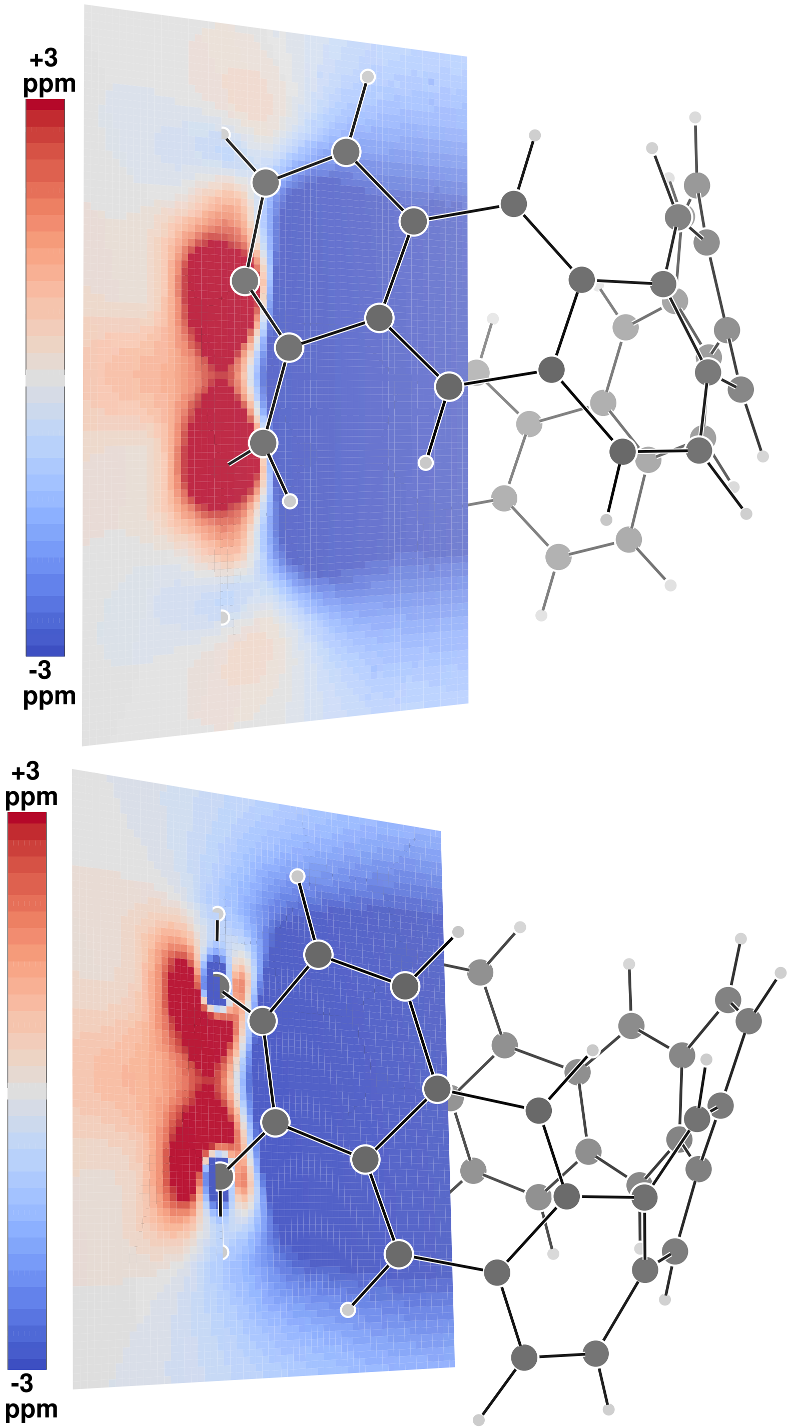

Figure S11: Divergent color maps of the contribution to σ_{zz} of **1** given by nine orbitals: HOMO, HOMO-1 ,..., HOMO-8. Top on a plane containing two rim protons; bottom on a plane containing bay protons. Planes extend from molecular axes toward the outside of the belt.

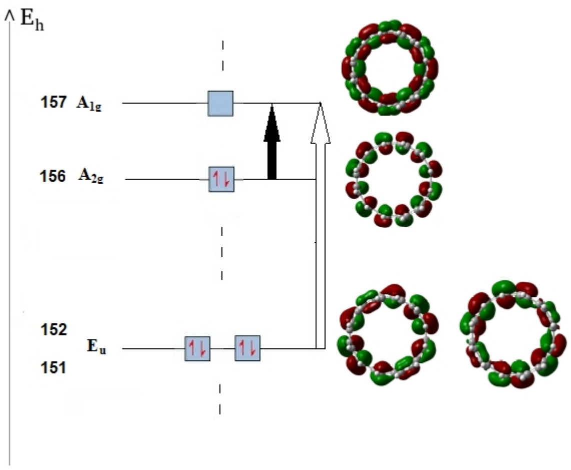

Figure S12: Contributions to total current density for **1** given only from frontier orbitals. White arrow indicate a diamagnetic current while black arrow a paramagnetic current.

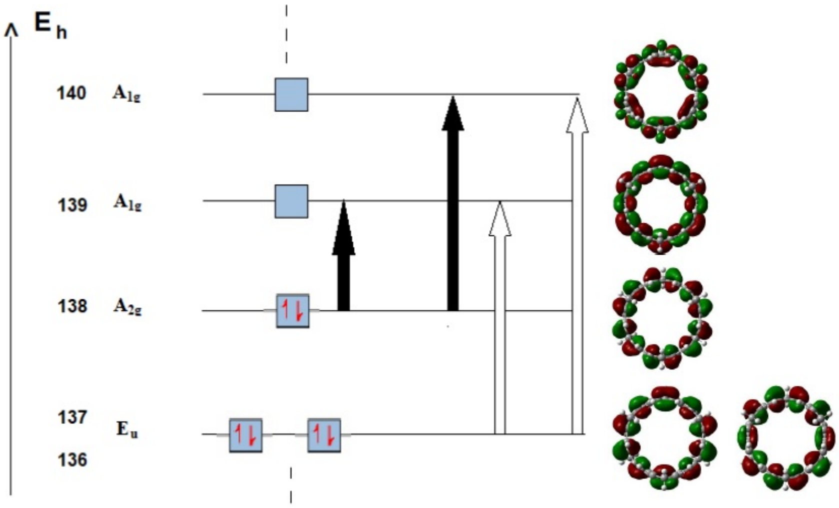

Figure S13: Contributions to total current density for **2** given only from frontier orbitals. White arrows indicate a diamagnetic current while black arrows a paramagnetic current.

References

[1] A. D. Becke, J. Chem. Phys. **98**, 5648 (1993).

[2] D. Flaig et al., J. Chem. Theory Comput. **10**, 572 (2014).

[3] R. R. Valiev, H. Fliegl, and D. Sundholm, Chem. Comm. **53**, 9866 (2017).

[4] R. R. Valiev, G. V. Baryshnikov, and D. Sundholm, Phys. Chem. Chem. Phy. **20**, 30239 (2018).

[5] M. J. Frisch et al., Gaussian 16, revision c.01, Gaussian, Inc., Wallingford CT, 2019.

[6] G. Monaco, F. F. Summa, and R. Zanasi, Chem. Phys. Lett. **745**, 137281 (2020).

[7] G. Monaco, F. F. Summa, and R. Zanasi, SYSMOIC package, University of Salerno, publication in progress, 2020.

[8] M. W. Lodewyk, M. R. Siebert, and D. J. Tantillo, Chem. Rev. **112**, 1839 (2012).

[9] H. E. Gottlieb, V. Kotlyar, and A. Nudelman, J. Org. Chem. **62**, 7512 (1997).

[10] J. Juselius, D. Sundholm, and J. Gauss, J. Chem. Phys. **121**, 3952 (2004).

[11] H. Fliegl, D. Sundholm, S. Taubert, J. Juselius, and W. Klopper, J. Phys. Chem. A **113**, 8668 (2009).

[12] G. Monaco, R. Zanasi, S. Pelloni, and P. Lazzeretti, J. Chem. Theory Comput. **6**, 3343 (2010).
